# A decade of antiretroviral therapy in Uganda: what are the emerging causes of death?

**DOI:** 10.1186/s12879-019-3724-x

**Published:** 2019-01-21

**Authors:** Agnes N. Kiragga, Frank Mubiru, Andrew D. Kambugu, Moses R. Kamya, Barbara Castelnuovo

**Affiliations:** 1 Research Department, Infectious Diseases Institute, College of Health Sciences, Makerere University, Kampala, Uganda; 2 School of Medicine, College of Health Sciences, Makerere University, Kampala, Uganda

**Keywords:** HIV, Antiretroviral therapy, Causes, Death, Mortality, AIDS

## Abstract

**Background:**

The roll out of antiretroviral therapy (ART) in Sub-Saharan Africa led to a decrease in mortality. Few studies have documented the causes of deaths among patients on long term antiretroviral therapy in Sub-Saharan Africa. Our objective was to describe the causes of death among patients on long term ART in Sub-Saharan Africa.

**Methods:**

We used data from a prospective cohort of ART naïve patients receiving care and treatment at the Infectious Diseases Institute in Kampala, Uganda. Patients were followed up for 10 years. All deaths were recorded and possible causes established using verbal autopsy. Deaths were grouped as HIV-related (ART toxicities, any opportunistic infections (OIs) and HIV-related malignancies) and non-HIV related deaths while some remained unknown. We used Kaplan Meier survival methods to estimate cumulative incidence and rates of mortality for all causes of death.

**Results:**

Of the 559, (386, 69%) were female, median age 36 years (IQR: 21–44), 89% had WHO clinical stages 3 and 4, and median CD4 count at ART initiation was 98 cells/μL (IQR: 21–163). A total of 127 (22.7%) deaths occurred in 10 years. The HIV related causes of death (*n* = 70) included the following; Tuberculosis 17 (24.3%), Cryptococcal meningitis 10 (15.7%), Kaposi’s Sarcoma 7(10%), HIV related toxicity 6 (8.6%), HIV related anemia 5(7.1%), *Pneumocystis carinii Pneumonia* (PCP) 5 (7.1%), HIV related chronic diarrhea 4 (5.7%), Non-Hodgkin Lymphoma 3 (4.3%), Herpes Zoster 2 (2.8%), other 10 (14.3%). The non-HIV related causes of death (*n* = 20) included non-communicable diseases (diabetes, hypertension, stroke) 6 (30%), malaria 3 (15%), pregnancy-related death 2 (10%), cervical cancer 2 (10%), trauma 1(5%) and others 6 (30%).

**Conclusion:**

Despite the higher rates of deaths from OIs in the early years of ART initiation, we observed an emergence of non-HIV related causes of morbidity and mortality. It is recommended that HIV programs in resource-limited settings start planning for screening and treatment of non-communicable diseases.

**Electronic supplementary material:**

The online version of this article (10.1186/s12879-019-3724-x) contains supplementary material, which is available to authorized users.

## Background

The roll out of antiretroviral therapy (ART) in Sub-Saharan Africa led to a decrease in mortality [1]. AIDS-related deaths have fallen by 48% since the peak in 2005 [2]. The UNAIDS 2016 global report shows gains in treatment of HIV positive persons were responsible for a 26% decline in AIDS-related deaths from an estimate of 1.5 million [1.3 million – 1.7million] persons in 2010, to 1.1 million [940,000–1.3million] in 2015 [3]. While in developed countries ART has been available for more than 20 years [4], in Sub-Saharan Africa ART has been available for slightly over a decade. The first countries to roll out ART, such as Malawi, begun in 2003 [5, 6]. In Uganda, ART was initially rolled out in 2004 and targeted severely immune compromised patients enrolled in HIV care [7].

The success of ART roll out in sub-Saharan Africa has been undermined by high mortality, especially in the first year after starting ART [8]. This high mortality was highest or similar to pre-ART mortality in the first months of ART and largely driven by opportunistic infections, especially tuberculosis, Cryptococcal meningitis and sepsis [8].

However, there is limited information on the specific causes of death among patients on long term ART in Sub-Saharan Africa. The recent audit of global deaths by the World Health Organization and Global Burden of Disease reported several causes of death, of which HIV was listed among the top 10 causes in 2006 [9]. In our work we aim to disaggregate this further to describe the causes of death among HIV positive persons in SSA. We also previously described mortality in only the first three years in a research cohort of patients started on treatment during the initial roll out of ART. Our results showed that the most common causes of early mortality were tuberculosis and Cryptococcal meningitis [10].

In this study, we sought to explore the trends and causes of death over time during the first 10 years of follow up among a well-characterized cohort of patients, with emphasis in estimating HIV-related and non-HIV related mortality rates. In addition, the causes of death described here are over a ten-year period, as many have been described among shorter duration. Information reported on causes of death will be useful for comparison with other African cohorts that rolled out ART around the same time. We hypothesized that there would be a decrease in HIV-related deaths over time.

## Methods

This is a prospective cohort study that enrolled a total of 559 HIV positive patients who were followed up for 10 years. The study was carried out at the infectious Diseases Institute (IDI) in Kampala, Uganda, a center of excellence for HIV care and treatment. Such centers of excellence in Africa have shown to provide environment and platforms that address a wide range of health care services [11]. At the start of national ART roll-out in 2004, the IDI provided ART to severely sick persons who were in need for immediate treatment. Details of this research cohort our have previously been published elsewhere [12, 13]. In brief, this closed cohort consisted of subjects ART eligible according to the WHO guidelines (ART initiation with CD4 < 200 cells/uL) [14], residing within 20 Km of Kampala, and willing to comply with the study procedures. Participant enrolment took place between April 2004 to April 2005. All patients reporting to the IDI clinic and met the inclusion criteria were consecutively enrolled until the desired number was attained. Patients were monitored and were expected to return for clinical evaluation every three months. They received laboratory monitoring that included 6-monthly CD4 and HIV RNA viral load assessments [13]. All patients who missed a scheduled visit were first contacted by phone to ascertain any clinical events, or, if this was unsuccessful, through a home visit. If found dead, the team used all available means to ascertain the cause and place of death.

During the ten-year period, all participant causes of death were determined by a study physician from a combination of source documents, including review of out-patient medical records such as laboratory results, in-patient hospital charts, and a structured interview with the patients’ next of kin (“verbal autopsy”). All patients who died were included in this analysis. Causes of death were defined as HIV-related if they were due to an ART toxicity, any opportunistic infection due to HIV or HIV-related malignancy). Non-HIV related deaths included other causes of death such as trauma, cancer, road traffic accidents and others.

### Statistical analysis

We used descriptive statistics to identify the demographic and clinical characteristics of the study population, and also evaluated the follow up status of the patients including the proportions of patients who died at the different years from ART initiation. We compared causes of death for the following study periods: enrollment to 1 year of follow up, 1 to 5 years of follow up, and 5 to 10 years of follow up. Patients were followed up from date of ART initiation to date of death, or loss to follow-up (LTFU) or at their last clinic 10-year in the cohort. We used Kaplan Meier survival methods to estimate cumulative incidence and rates of mortality for all causes of death, non-HIV related conditions, HIV-related opportunistic infections and malignancies and toxicity. The Kaplan Meier has the following; each participant must be independent and appears once in their group, groups must be independent, all participants are event free when they enroll in the study, measurement of time to event is precise and start and events are clearly defined. The above assumptions were met in our study. All patients appeared in one group, were initiated on ART and alive at start of study therefore event (death) free, we ascertained the start of ART initiation as our start of risk, and the event of death was ascertained during patient follow-up. The case fatality rates for the most common opportunistic infections were also obtained as the percentage of patients classified as cases who died of the specific disease. The analysis was performed using STATA 14.1, Texas, USA.

## Results

A total of 559 HIV positive patients were included in the analysis, of whom majority were female (386, 69%) with median age of 36 years (interquartile range (IQR): 21–44), 89% were in WHO clinical stages 3 and 4 (89%), with a median CD4 count of 98 cells/μL (IQR: 21–163). The majority (414 (74%)) of the patients were started on Stavudine plus lamivudine plus Nevirapine and 145 (26%) on zidovudine plus lamivudine plus Efavirenz. After the first year of ART 83.2% (465) of patients had been retained in care, 385(68.9%) at 5 years, and 63.3% (354) at 10 years (see Additional file 1: Figure S1). The median follow-up time was a total of 3694 person-years and a total of 127 (22.7%) deaths occurred during the ten years of follow-up. A total of 70/127 (55.1%) were HIV-related, 20 (15.7%) were non-HIV related, and 37 (29.1%) had unknown causes of death. Eighty (63%) of the deaths occurred with the first year of ART initiation. A total of 354 patients were alive and active in follow-up after 10 years from cohort inception.

### Causes of death

The HIV related causes of death (*n* = 70) included the following; Tuberculosis 17 (24.3%), Cryptococcal meningitis 10 (15.7%), Kaposi’s Sarcoma 7(10%), HIV-related toxicity 6 (8.6%), HIV related anemia 5(7.1%), *Pneumocystis carinii Pneumonia* (PCP) 5 (7.1%), HIV related chronic diarrhea 5 (5.7%), Non-Hodgkin Lymphoma 3 (4.3%), Herpes Zoster 2 (2.8%), other 10 (14.3%). The non-HIV related causes of death (*n* = 20) included non-communicable diseases (diabetes, hypertension, stroke) 6 (30%), malaria 3 (15%), pregnancy-related death 2 (10%), cervical cancer 2 (10%), trauma 1(5%) and others 6 (30%).

**Fig. 1:**
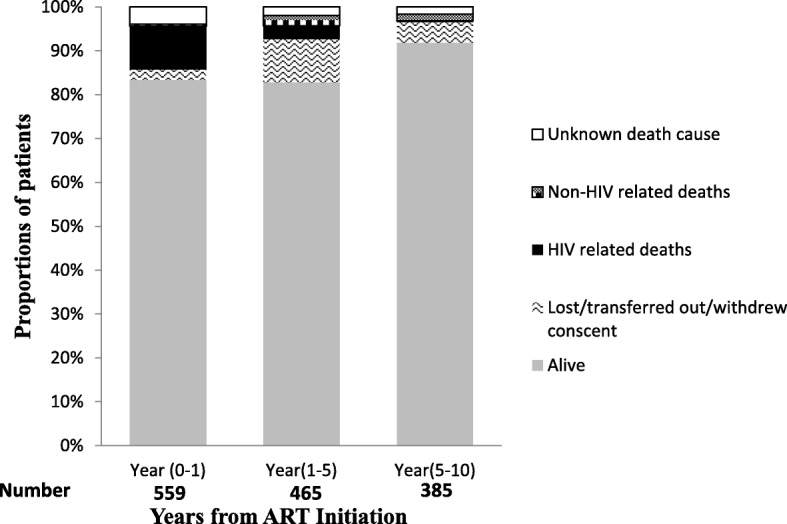
Follow-up status and cause of death during 10 years among HIV naïve who initiated Antiretroviral Therapy in Kampala, Uganda

Figure 1 summarizes the patients’ follow up status and category of death among patients after ART initiation. In the first year of ART, out of 559, we observed 55 (9.8%) HIV-related deaths, 3 (0.5%) non-HIV related. At end of the first year, 465 patients were alive and in care, and of these 14 (3.0%) later died of an HIV-related cause and 11 (2.4%) non-HIV related. At the end of 5 years from ART initiation, 385 patients were still alive and in care, and of these 1 (0.3%) died due to an HIV-related death and 6 (1.6%) died of a non-HIV related cause. At the end of our study follow-up (10 years), 354 patients were alive and in care. We observed a trend towards reduction in the proportion of patients with HIV-related causes (including ART toxicity, opportunistic infections and HIV-related malignancies) of death overtime from 9.8% in years to 0.3% *P* value for test for trend, *P* < 0.001. We also observed a modest increase in the non-HIV related causes of death from 0.5 to 1.6% in the later years.

### Mortality rates

**Fig. 2:**
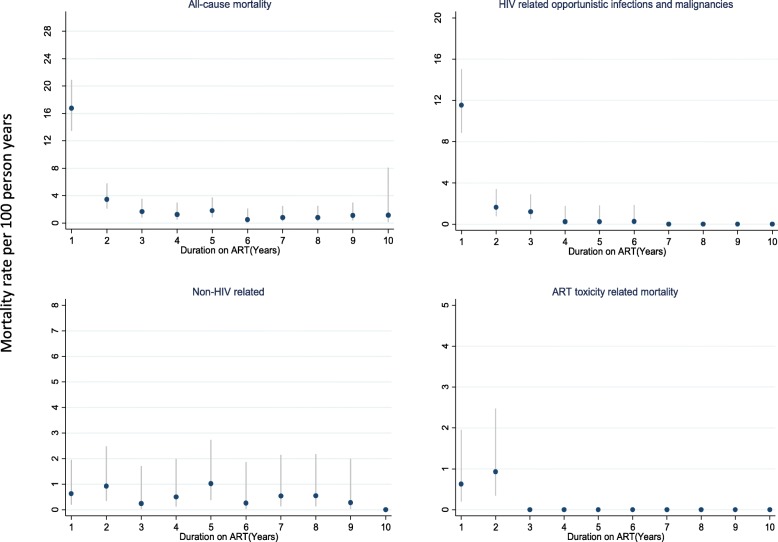
Cause specific mortality rate per 100 person years of follow-up among HIV naïve patients who initiated ART in Kampala, Uganda

The cumulative rate of all causes mortality in the first year was 16.8 (13.4–20.9) per 100 person years of follow-up, 1.8 (0.9–3.9) at year 5, and 1.1 (0.2–8.1) at year ten (Fig. 2). At year 1, 5 and 10, the mortality rate among females was 15.7 (12.0–20.6), 1.8 (0.8–4.4) and 1.6 (0.2–11.6) compared to 19.1 (13.2–27.7), 1.8 (0.4–6.7) and 0.0 (0.0–0.0) per 100 years of follow-up among males.

The mortality rates per 100-person years of follow-up at years 1, 5 and 10 for AIDS related deaths was 11.5 (8.8–15.0), 0.3 (0.0–1.8), 0.0 (0.0–0.0) compared with 0.6 (0.2–2.0), 1.0 (0.4–2.0), 0.3 (0.1–2.0) for non-HIV related deaths. As expected, mortality rates due to toxicities alone reduced from 0.6 (0.2–1.9) per 100-person years within the first year of ART, to none in years 1–5 and 5–10. cryptococcal meningitis, tuberculosis and Kaposi’s Sarcoma were the most common causes of death among the observed opportunistic infections. The case fatality rates for the three most common opportunistic infections Cryptococcal Meningitis, tuberculosis and KS were obtained. A total of 20 persons were diagnosed with Cryptococcal meningitis and of these 10 died, yield a case fatality rate of 50%. Similarly, 63 persons were diagnosed with Tuberculosis, and of these 16 were confirmed dead, yielding a case fatality rate of 25%. And lastly, 7 patients among the 10 died diagnosed with Kaposi’s sarcoma died from the disease yielding a case fatality rate of 70%.

## Discussion

Our study describes the causes of death overtime up to 10 years of follow up among HIV positive patients in Sub-Saharan Africa. We observed 127 (22.7%) deaths among the patients who initiated ART, and majority (55%) were HIV related deaths. The majority (63%) of deaths were observed within the first year due to the very low immune suppression of the patients at the roll out of free ART. HIV-related deaths contributed to the bulk of the observed deaths at 55%, while 20 (15.7%) were non-HIV related, and 37 (29.1%) could not be ascertained. Overtime the proportion of HIV related deaths decreased from 9.8 to < 1% at ten years. Tuberculosis, Kaposi’s sarcoma and Cryptococcal meningitis were the most commonly occurring opportunistic infections with high case fatality rates.

Our results are similar with previous reports. The recently WHO global burden disease highlights HIV as one of the top 10 global causes of death [9, 15]. A study in Ethiopia showed similar contribution of HIV related deaths, with 85% of all deaths after ART initiation occurring in the first year [16]. The general trend in reduction of HIV related mortality over a ten year period was reported in the USA [17], and in South Africa where a 10% reduction in HIV morality was observed [18]. Similarly, a 70% reduction in HIV-related mortality was observed in a period of 15 years [19]. Tuberculosis and Cryptococcal meningitis have similarly been highlighted among the leading causes of HIV-related deaths in Sub-Saharan Africa accounting for 15–30% of deaths after ART initiation [20–23]. In other parts of Sub-Saharan Africa, the increase in deaths due to non-communicable diseases (NCDs) has been noted. In Ethiopia and other sub-Saharan countries, NCDs are becoming a leading cause of death [19]. Our findings general concur with previous studies and contribute to the body of literature on causes of death during the first decade of ART rollout in Sub-Saharan Africa.

In sub-Saharan Africa this high HIV related mortality was driven by severe immunosuppression of our patients and therefore predisposition to opportunistic infections [24, 25]. Following ART initiation a robust immune recovery leads to over 50% of the patients reaching a CD4 count of 400 c ells/ml by year 5 [13]. Consequently, the reduction in mortality is then driven by the decrease in opportunistic infections and HIV related malignancies. In our study the decrease in mortality was also attributed to the reduction in toxicity related mortality. Three quarters of patients were started on stavudine-containing regimens which were associated with several toxicities. Following the Uganda Ministry of Health recommendations in 2008, all patients on stavudine containing regimen were switched to zidovudine or tenofovir containing regimens.

We observed a modest increase non HIV-related deaths. Factors contributing to non-HIV related mortality included increasing age, lifestyle factors, as well as chronic inflammation and immune activation [26–28].

Our study had several limitations, the primary one being the lack of verbal autopsy. We acknowledge that this might have contributed to minimal misclassification of causes of death and led to an outcome ascertainment bias. The study team used all possible means to obtain the causes of death from structured interviews over the phone and or home visits. Based on the information, the study physician made a diagnosis for the causes of death. Generally, the ability to diagnose etiologic agents of death in our setting is limited and, 30% of deaths were unknown. Current verbal autopsy methods underestimate mortality due to HIV-associated TB [29]. Even though the availability of more robust and accurate autopsy methods have increased over time, however, there exist barriers to acceptability of autopsies in our setting. A study carried out by colleagues at the national referral hospital in Uganda showed only 38% of next of kin accepted autopsies and reasons for refusals included next of kin seeing no need to know the exact cause of death, generally being satisfied with the clinical cause of death and believing in other cultural beliefs surrounding deaths [30].

Secondly, we had limited information on underlying comorbidities at ART initiation that could have confounded the observed mortality such as previous blood transfusions. However, like in many sub-Saharan African countries, sexual transmission remains the most common means of acquiring HIV.

Additionally, the observed trends and causes of mortality might not reflect the current state in the era of Test and start. The early roll –out of ART was faced with severely immune suppressed patients. The type of antiretroviral drugs prescribed have also changes over time. Also, the criteria for ART initiation based on CD4 was progressively lifted from 200 to 350, then to 500 cells/μL and finally to the current recommendation of starting regardless of CD4 count (“test and treat”) [31]. Our results maybe generalizable to similar settings in Sub-Saharan Africa with limited resources available for post mortem services and in settings where cultural beliefs over-ride the need to have the exact cause of death obtained.

## Conclusion

In conclusions our findings suggest a general reduction in mortality after ART initiation, and particularly a reduction in HIV related mortality. With the scale up of ART, HIV positive persons continue to life longer with an increase in life expectancy [32]. Traditionally, where resources are available, emphasis in our settings is put in the screening of opportunistic infections. However, with the emerging of non-HIV related causes of morbidity and mortality, it is recommended that HIV program in resource-limited settings start planning for screening and treatment of non-communicable diseases. Understanding the causes of death in this era of test and start is critical. We recommend future research on the magnitude of mortality and morbidity among late presenters or patients who drop out and re-engage in care.

## Additional file

Additional file 1: **Figure S1.** Infectious Diseases Institute research cohort profile in 10 years of follow-up between April 2005 and April 2015. The figure shows the number of patients enrolled in research cohort and the numbers of those who remained care, were dead, transferred-out or lost to follow-up during the 10 years of follow-up. (PDF 126 kb)

## Abbreviations

ART: Antiretroviral therapy
HIV: Human Immune deficiency Virus
KS: Kaposi’s Sarcoma
LTFU: Loss-to follow-up
NCD: Non-communicable disease
OI: Opportunistic infection
PCP: Pneumocystis carinii pneumonia
SSA: Sub-Saharan Africa
TB: Tuberculosis
USA: United States of America
