# Supplementary figures and images for: A decade of antiretroviral therapy in Uganda: what are the emerging causes of death?
Source: BMC Infect Dis. 2019 Jan 21;19:77. doi: 10.1186/s12879-019-3724-x (PMC6341568; doi:10.1186/s12879-019-3724-x)

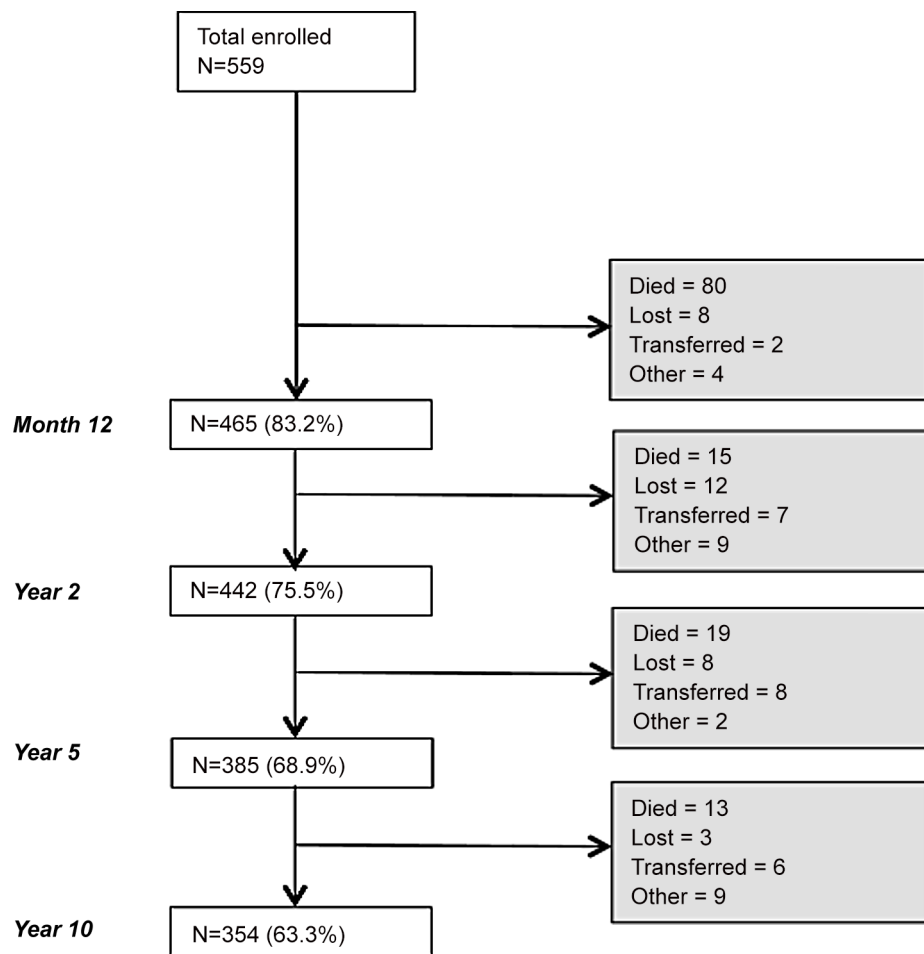

Supplement: Supplementary file 1 — Figure S1. Infectious Diseases Institute research cohort profile in 10 years of follow-up between April 2005 and April 2015. The figure shows the number of patients enrolled in research cohort and the numbers of those who remained care, were dead, transferred-out or lost to follow-up during the 10 years of follow-up. (PDF 126 kb) [file 12879_2019_3724_MOESM1_ESM.pdf]
